# Extending access to essential services against constraints: the three-tier health service delivery system in rural China (1949–1980)

**DOI:** 10.1186/s12939-017-0541-y

**Published:** 2017-05-23

**Authors:** Xing Lin Feng, Melisa Martinez-Alvarez, Jun Zhong, Jin Xu, Beibei Yuan, Qingyue Meng, Dina Balabanova

**Affiliations:** 1 School of Public Health, Peking University, Xueyuan Road 38#, Beijing, 100191 China; 2 London School of Hygiene and Tropical Medicine, London, UK; 3 Peking University China Center for Health Development Studies (CCHDS), Beijing, China

**Keywords:** Health service delivery, Health system, China, Resource scarce settings

## Abstract

**Background:**

China has made remarkable progress in scaling up essential services during the last six decades, making health care increasingly available in rural areas. This was partly achieved through the building of a three-tier health system in the 1950s, established as a linked network with health service facilities at county, township and village level, to extend services to the whole population.

**Methods:**

We developed a Theory of Change to chart the policy context, contents and mechanisms that may have facilitated the establishment of the three-tier health service delivery system in rural China. We systematically synthesized the best available evidence on how China achieved universal access to essential services in resource-scarce rural settings, with a particular emphasis on the experiences learned before the 1980s, when the country suffered a particularly acute lack of resources.

**Results:**

The search identified only three peered-reviewed articles that fit our criteria for scientific rigor. We therefore drew extensively on government policy documents, and triangulated them with other publications and key informant interviews. We found that China’s three-tier health service delivery system was established in response to acute health challenges, including high fertility and mortality rates. Health system resources were extremely low in view of the needs and insufficient to extend access to even basic care. With strong political commitment to rural health and a “health-for-all” policy vision underlying implementation, a three-tier health service delivery model connecting villages, townships and counties was quickly established. We identified several factors that contributed to the success of the three-tier system in China: a realistic health human resource development strategy, use of mass campaigns as a vehicle to increase demand, an innovative financing mechanisms, public-private partnership models in the early stages of scale up, and an integrated approach to service delivery. An implementation process involving gradual adaptation and incorporation of the lessons learnt was also essential.

**Conclusions:**

China’s 60 year experience in establishing a de-professionalized, community-based, health service delivery model that is economically feasible, institutionally and culturally appropriate mechanism can be useful to other low- and middle-income countries (LMICs) seeking to extend essential services. Lessons can be drawn from both reform content and from its implementation pathway, identifying the political, institutional and contextual factors shaping the three-tier delivery model over time.

## Background

Since the establishment of the People’s Republic of China in 1949, the country has experienced major socio-economic changes; the population has more than doubled and the country has progressed from low to upper-middle-income status, according to the World Bank classification [1]. This has been accompanied by dramatic increases in the number of health facilities and human resources, and improvements in the accessibility to medicines and medical supplies. For instance, the number of health care providers increased 260-fold between 1949 and 2011 [2]. Consequently, preventive, curative, rehabilitative and palliative care has become increasingly available and accessible [3]. A three-tier health system, established as a network connecting health service facilities at county, township and village level, was established in the 1950s with the aim of extending services to the whole population [4]. The World Health Organisation (WHO) has recognised the Chinese three-tier system as one of the “three magic weapons” to provide universal primary health care (PHC) [5], the other two being the primary care doctors with basic level of training, known as barefoot doctors, and the cooperative medical scheme (the predecessor of the current new rural cooperative medical scheme). The architecture of the three-tier system reflected many of the Alma Ata Declaration principles such as providing comprehensive community-oriented PHC, “health-for-all” based on participation and the right to better health [6]. The Chinese experience of implementing these principles has inspired a range of initiatives towards developing comprehensive and inclusive primary care, and many local adaptations in low- and middle-income countries (LMICs) [7–10].

Despite this progress, the reforms were reversed in the 1980s, with a move towards market-based structures and liberalisation that changed the relationship between the different levels of providers and shifted the balance of provision from primary health care to tertiary highly specialised care, often at high cost to the users [11, 12]. Following this, since 2009 there has been a renewed effort to reorient services towards primary health care and encourage PHC utilisation, reviving many of the principles from the pre-1980s community-based integrated PHC model [13, 14].

Given these policy shifts, it is important to reflect on the six-decade evolution of China’s rural health service delivery system, and draw lessons for other LMICs seeking to progress towards universal coverage, through PHC strategies. The Chinese experience with the three-tier service delivery model as a vehicle to deliver essential care, has been evoked in global debates around universal health coverage as an example of a country able to achieve significant advances despite lower level of economic development in its early stages [11]. In addition, some central and eastern European countries that adopted a similar Soviet model of health care are currently undergoing a transition in their health system transition and may find useful lessons from the development of the Chinese three-tier health system [14]. However, the features of this model, its development over time, and the factors enabled it, and its subsequent adaption, have not been well documented in international publications.

This paper seeks to address this gap by systematically reviewing and synthesising the current knowledge on the foundation, organization and evolution of the three-tier health service network in rural China. The policies and interventions are explored within their historical context, as a critical lens for understanding their design and implementation. Specifically, we aim to answer the following questions: (i) how did China establish the three-tier service delivery system? (ii) what were the characteristics of the system and what kind of health services were offered to the rural population? (iii) what factors made the three-tier system successful, and (iv) what were the implications of the structural changes of the China’s rural health service delivery system within the rapidly evolving economic, social and political context, for the availability, efficiency, quality and equity of essential care?

## Conceptual framework

The research approach was underpinned by a theory of change (ToC) drawing on realist evaluation principles [15]. The ToC was developed by the authors in collaboration with a broader team of global health experts during two workshops held in London and Beijing in 2015. The purpose of the ToC was to identify the policy content related to the three-tier health system, the outcomes associated with its expansion, to outline plausible mechanisms through which outcomes were achieved, and to identify the contextual factors that facilitated the development and implementation of this delivery model.

**Fig. 1:**
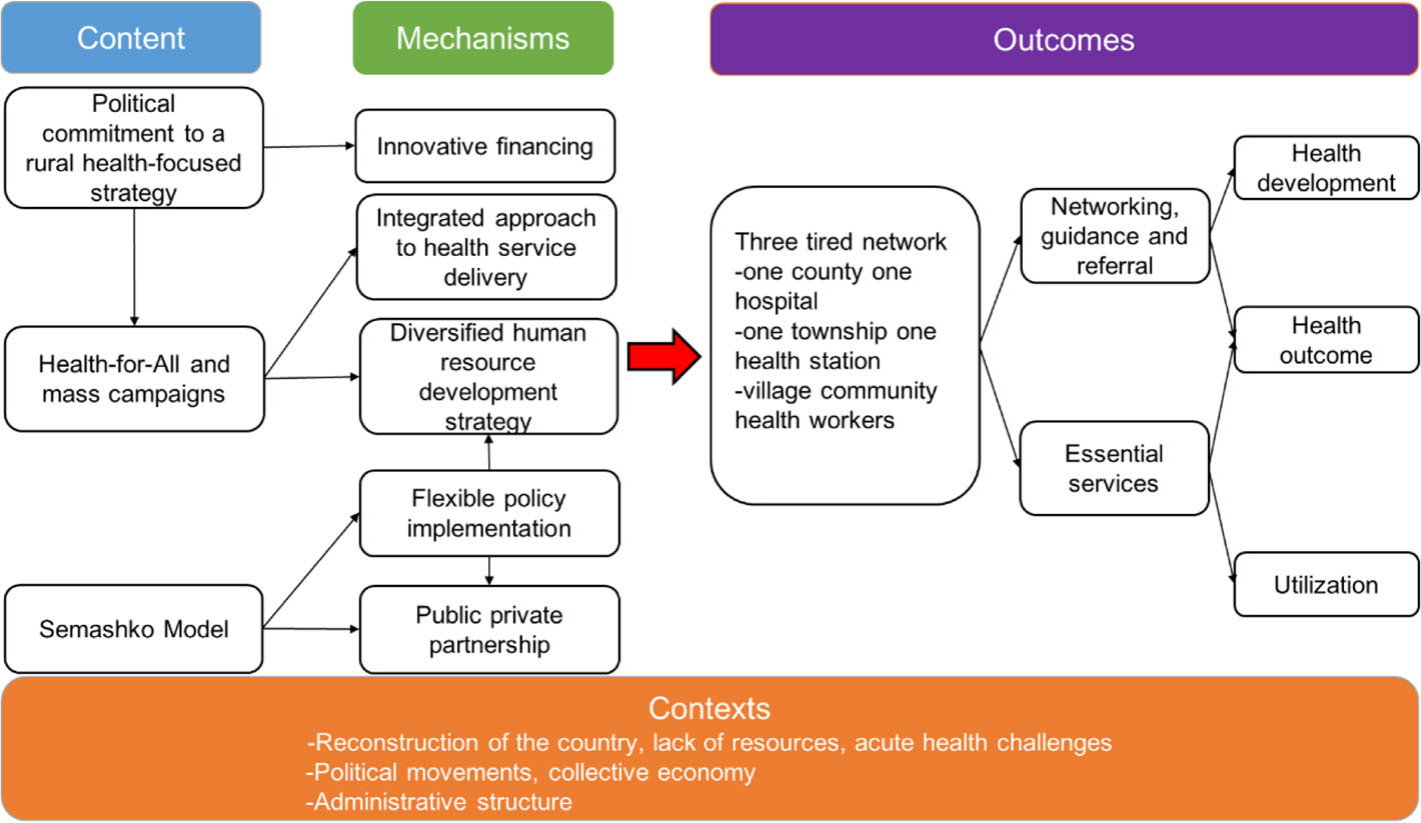
Theory of Change on the establishment of the three-tier health system

Our ToC is shown in Fig. 1. It develops a set of context, mechanism and outcome result chains by linking policy content (input), processes, contextual factors and outcomes. The expert workshops identified a range of potentially important political and socio-economic contextual factors. Through the analysis we were able to identify those that played a role in the design and implementation of the three-tier system, including availability of health resources, and the Chinese political, economic and administrative structure. In terms of the content of policies that led to the establishment of the three-tier system we included the Communist party’s political endorsement of rural health, a health-for-all policy vision to guiding mass campaign movements, and the adoption of Soviet Semashko model as an organization structure of the health system. In terms of mechanisms, following from the expert workshops and the literature review we synthesized five different mechanisms through which the three-tier system was established, including human resource development strategies, integration of existing health service structures, health financing, public-private partnerships, and a flexible approach to policy implementation. Lastly, we included a range of intermediate and long-term outcomes, including the health system characteristics, utilization of care and health outcomes.

## Methods

We systematically searched for articles providing evidence related to each of the dimensions of our ToC, with a focus on historical papers addressing the time period of 1949–1980. It was used as an analytical framework to guide the process of publication selection and interpretation. Data were extracted for each dimension of the framework, while seeking to capture the history and sequence of implementation. The ToC was updated and reframed iteratively as the analysis progressed.

We searched publications from peer-reviewed journals, books and grey literature, including Masters/PhD dissertations and policy documents, that report on the foundation, evolution, and functioning of the China’s rural three-tier health system. First we searched for peer-reviewed articles in the following databases: PubMed, Web of Science, EMBASE, Scopus, WHOLIS, China Knowledge Resource Integrated Database (CNKI), and Google Scholar. We then searched for grey literature using the ProQuest Dissertation & Theses Database and the Wanfang Dissertation Database as well as policy archives and unpublished manuscripts provided by leading experts in the field. Snowballing was subsequently used to identify further relevant papers.

The following search terms were used: China, rural, health service*, health delivery, health care, healthcare, health system*, three-tier, health, village clinic*, village post, barefoot doctors, bare-foot doctors, township health cent*, township hospital*, county hospital*, community health cent* and community health station*.

**Fig. 2:**
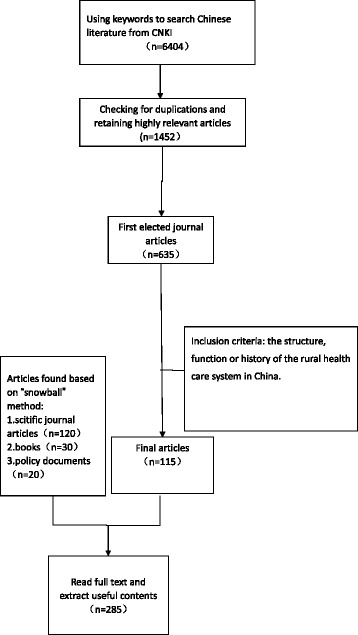
Searching process for Chinese literature

**Fig. 3:**
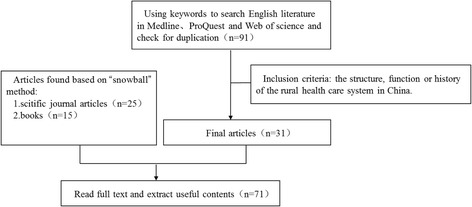
Searching process for English literature\

The lead author and two co-authors (JZ and JX) screened all the publications by title. Two researchers JZ and JX then independently screened the abstracts and discussed any discrepancies in the assessment with the lead author to reach consensus. Publications reporting on the structure, function or history of the rural health care system in China were included. We included all study designs. Papers reporting primarily management experiences without information on institutional structures, functions or historical analyses were excluded. This process led to a total of 285 Chinese and 71 English publications (see Figs. 2 and 3, PRISMA chart).

We then appraised the quality of the selected articles, in terms of relevance to the study questions and theory of change. With the exception of three anthropological peer reviewed papers (Wang [16], White [17], and Huang [18]), most of the publications were either descriptive in nature, or represented the authors’ observations or opinions rather than finding from empirical research. Drawing on the principles of realist synthesis [15], we sought to take a historical perspective [19], demonstrating how policies and practices develop within their historical context. We drew on government policy documents to trace the development and evolution of the policies and programmes. Information retrieved from the central governments’ documents archives and statistics [2, 20] was triangulated with the anthropological studies and other relevant analyses and observations (including Rifkin, Sidels, Horns, and Rosenthal, Hsu, Wen, New, Yang etc. [7–9, 21–26],) and the relevant publications in Chinese journals based on local observations [27, 28]. Our review showed that these sources of evidence were generally consistent in analysing the historical events and forces triggering the establishment and evolution of the three-tier system.

Findings were further validated through series of consultations with a leading expert on the Chinese three-tier system. Dr. Zikuan Zhang, born in 1929, is considered to be one of the founders of China’s rural health service system [16]. He was the former director in charge of health service management in the Ministry of Health before his retirement and has had a major involvement in all the major reforms in 1949–1991.

## Results

Results are presented following the four components of the ToC framework: how political and socio-economic processes facilitated or obstructed the establishment of particular types of policies (context), the main policies and their operationalisation to a set of concrete policies (policy content), how these policies were implemented in practice and what factors made them work as intended (mechanisms) and shaped the establishment of the three-tier health service delivery system in rural China as a key vehicle for extending access to PHC (outcomes).

### Context

#### Political context

**Fig. 4:**
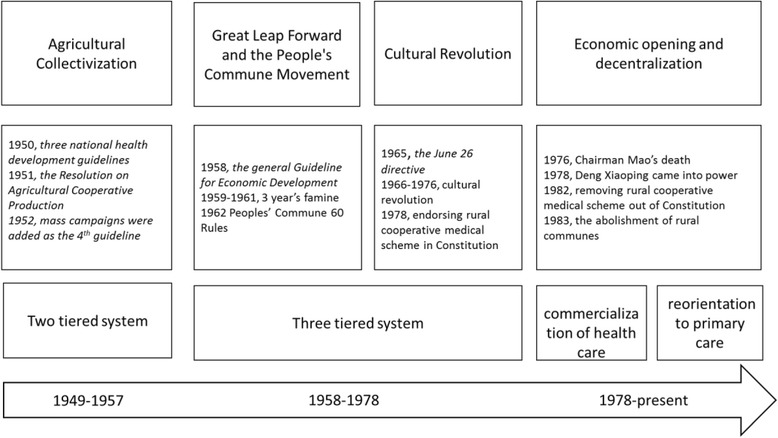
Major socio-political events and health system evolution in China since 1949

Historically, the evolution of the political, economic and administrative institutions in rural China can be divided into three periods (Fig. 4): the Agricultural Collectivization period (1949–1957), the People’s Commune period (1958–1978, encompassing the Great Leap Forward and the Cultural Revolution), and the Economic Opening and Decentralization period (1978-present) [29]. The Agricultural Collectivization period began during the establishment of the People’s Republic of China, when the country was recovering from a prolonged period of war (8 years of war against Japan and 3 years of civil war between the Communist Party of China (CPC) and the Guomin Dang).

The country initiated reconstruction with extremely scarce resources in a context of very poor socio-demographic characteristics. Life expectancy was 35 years on average, with the major health challenges including high prevalence of infectious diseases, and high maternal and under-five mortality (maternal mortality 1500 per 100,000 live births and infant mortality 200 per 1000 as estimated in 1949) [2, 7–9, 30]. Health facilities and human resources were scarce and unevenly distributed, mainly concentrated in the urban areas. There were only 1400 county hospitals in more than 2200 counties nationwide, in addition to some church-run and military hospitals [4]. In rural areas, the density of hospital beds was 0.05 per 1000 population, with a few private facilities available (0.73 per 1000 population) [2], and there were acute shortages of medicines. According to Horn (1972) there was less than one doctor (trained in western medicine) per 100,000 people, and these were largely concentrated in major coastal cities and provincial capitals. Although the number of traditional doctors was higher, they also tended to not live in villages [7].

In September 1951, the CPC announced the Resolution on Agricultural Cooperative Production, promoting the formation of agriculture collectivization (the organization of farmers to form “collective ownership agricultural cooperatives”) to improve economic efficiency [31]. By the end of the period of Agricultural Collectivization, 96.3% of farmers were “organized” into agricultural cooperatives [4].

In 1958, the general Guideline for Economic Development was announced with the aim of achieving “faster, better and more economical results in building socialism” [31]. Following that, the Great Leap Forward was initiated as a national political movement to promote further collectivization for the rural farmers, with its implementation platform, the People’s Commune Movement, scaled up nationwide. Under these initiatives, communes were formed at the township level by merging various village agricultural cooperatives. The rapid consolidation process contributed to the 1959–1961 famine, causing starvation for millions of people [32].

National economic policy changed course with the enactment of the ‘Peoples Commune 60 Rules’ in 1962, decentralizing the ownership of the communes to village production teams. [31] In 1966, with the gradual recovery of the China’s economy, a growing social movement transformed into a political movement against the CPC leadership. This led to the start of the “Cultural Revolution”, re-imposing Maoist thought as the dominant ideology within the CPC and marking the return of Mao Zedong to leadership role. The return of Mao has been associated with a negative impact on the development of the country [33]. Interestingly, according to Wang, Sidels, White and Huang et.al, the rural areas benefited in terms of health development during this period [8, 16–18].

Mao’s death in 1976 brought the Cultural Revolution to an end. In 1978 the central leadership changed and the Chinese government implemented a strategic shift resulting in economic decentralization reforms. The collectivist economy was gradually dismantled and privatization of land and property took place in the rural areas, with the communes collapsing and farmland rented out to rural households [11, 34].

#### Administrative structure

The administrative structure in China was shaped by the country’s history and political development, and consisted of a six-level hierarchy: state, province, city, county, township, and village. Although the numbers varies historically, in general there were about 30 provincial level units, governing a total of around 300 cities, 3000 counties, 60,000 townships and 734,000 villages [35]. County and lower level divisions are typically referred to as rural areas [35]. In general, health planning was conducted at the provincial level while financing of health facilities were determined at county level. Transfers from central and provincial government were made to subsidize county level health care.

**Fig. 5:**
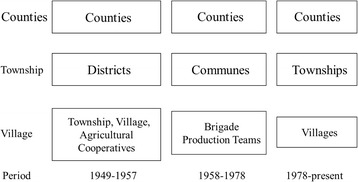
The administrative structure of rural China over the three historical periods

As shown in Fig. 5, the names of the various rural unit have changed over time. For consistency with the historical nomenclature, when describing divisions below the county level, we refer to “district”, “townships”, “villages” and “agricultural cooperatives” for the 1949–1957 period; “communes”, “brigades” and “production teams” for the 1958–1978 period; and “townships” and “villages” for the period from 1978 to the present.

### Policy content

#### Political commitment to a rural health-focused strategy

The three-tier health service delivery system was established in rural China shortly after the CPC united mainland China and came into power. The prioritization of health in the rural areas was key to the overarching government policy. Indeed, focusing on rural areas and farmers was a key strategy for the CPC to achieve and consolidate the regime and its leadership role. For example, in 1928 Mao defined the CPC’s political strategy of the revolution as promoting the “countryside encircles the cities”, identifying farmers living in the countryside as key constituency and the support base enabling the CPC to come into power [33]. After the World War II, the CPC concentrated its political and organizational resources to the countryside in north-eastern and western China in order to mobilize the rural masses. Further, the CPC supported farmers to set up agricultural cooperatives, which laid a solid political and economic foundation for the CPC to mobilize soldiers and supplies to establish the People’s Republic of China in 1949 [36].

#### Health-for-All and mass campaigns

The prioritization of health in the rural areas was due not only to political considerations, but also to the fact that CPC leaders regarded health as an important and integral part of a programme to promote the country’s social and economic development [7]. A focus on health improvement was considered a strategy to build a productive workforce. As Mao stated, “Health care is important, because it is in favour of production, in favour of work and in favour of study… (It) serves the majority of the people” [37]““Raising health status of the people” not only means “(providing) positive prevention and curative care, promoting the nation’s health development”, but also “eliminating all obstacles to achieve people’s good health”. The way to achieve these goals was to “mobilize the masses, rely on the masses and integrate health services with work and production.” [38] This “Health-for-All” [Multisectoral approach in Chinese language] concept effectively guided the implementation of China’s rural health policy through an integrated vision of health and development at the core of social policy.

Consequently, clear priority was given to rural health in China’s national health policy [33]. The policy was operationalised in the first National Health Conference in 1950, where the following aims of the national health system were established: (i) to “serve the needs of workers, peasants, and soldiers- wherever they happen to be”, (ii) to give priority to health prevention and (iii) to “integrate western and traditional Chinese medicine” [8, 38]. In 1952, the fourth principle, “achieving health development through mass campaigns” was further formulated [7–9, 16]. In 1965, in the “June 26th directives” Mao stated that “in health work put stress on the rural areas”, illustrating the unprecedented importance given to rural health, thus creating the political foundation for the establishment of the three-tier system for service delivery throughout China [7, 9].

Targeting and mobilizing the masses was the main strategy for health development activities, due to extreme shortage of health workforce [7, 39, 40]. Large-scale mass campaigns were implemented to enhance health promotion, including health education and vaccination promotion at festivals, markets, schools and other centres where the population gathered naturally [16, 41].

The process of health system development involved effectively integration of the various levels of health organizations, of existing resources and institutions, and of different types of health care (e. g. traditional Chinese and Western medicine), which brought most civil society institutions and various medical associations into a common policy framework. Literate farmers were trained to undertake primary health care services. Clean delivery—lying down for delivery and disinfecting mother’s perineum, scissors cutting the cord and the attendants’ hands—was also promoted through mass campaigns [16].

#### The adoption of the Semashko model as a prototype of a health system

Similar to the country’s administrative and organizational structure, China’s health system and health organizations replicated some of the principles of the Soviet Union Semashko model [42]. The three-tier health system was designed to fit the existing three level rural administrative structure. Similarly to the Semashko model, health planning and resource allocation took place at different administrative levels [42]. This meant that each administrative level had different levels of planning and managerial responsibilities related to oversight, management, guidance and supervision, information reporting, and accountability. Thus, health policies developed by the central government were implemented at the lower levels, and results reported back, guiding further policy adaptation. The alignment of the health service network with the respective geographical administrative levels, may have resulted in lower administrative costs of health planning [43]. The continuity of health reforms contributed to improving the structure and capacity of the management system, and evolved in line with the changes of the broader administrative structures over time.

This structure was meant to facilitate the core objectives of the Semashko model—improving access, equity, and public participation (in what can be seen as people centred perspective)—ensuring that all levels contribute to these goals. While the Semashko model was a traditionally centralised model with policy formulated at the top, in China it was implemented through a decentralised approach. The local governments were empowered to plan and manage the health systems with considerable autonomy and flexibility. Provincial, municipal and county level governments were granted much of the decision-making power of implementation so that they could set up their own priorities according to their own fiscal situation and local health priorities.

This reflected the aforementioned principle of “serve the needs of workers, peasants, and soldiers- wherever they happen to be” [7, 9], demonstrating a commitment to responsiveness. Thus, local governments were also able to adjust the allocation of health resources based on where the people were located, reflecting the perspective of people-centred health system planning. The central leaders reaffirmed the need to improve accessibility and PHC facility coverage—with many resemblances to the Soviet Union facing the challenges of large territory and dispersed population—by establishing extensive facility network located in proximity to the people [16, 33], with the aim of improving agricultural productivity [44, 45]. Therefore although township hospitals were usually set up according to the location of the township government, they were not limited to this administrative location. Some authorities even tried to set up health stations near the farming areas where the agricultural workers cultivate and harvest, reflecting the policy that there should be three services provided to the agricultural workers: human resources, medical supplies and health promotion and information [7, 8, 16, 18]. The three stages of implementation of the Chinese Semashko model are discussed below.

**Fig. 6:**
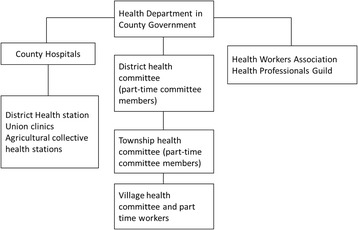
Structures of the rural service delivery system in the Agricultural Collectivization period

**Fig. 7:**
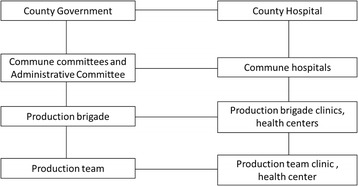
Structures of the rural service delivery system in the People’s Commune Period

**Fig. 8:**
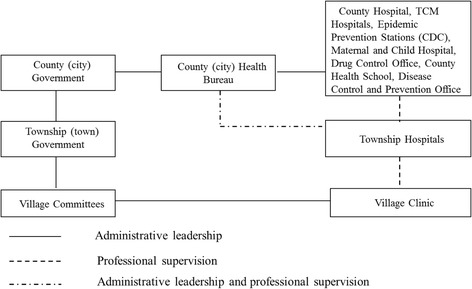
Structures of the rural service delivery system in the Economic Opening and Decentralization Period

1. The Agricultural Collectivization Period (1949–1957)
  This period was characterised by prioritising public health and PHC over curative care. The priority was to establish a service network organised in two tiers, at the county and township level, through a mix of government-owned facilities and public-private partnerships (Fig. 6). At the county level, a government-owned county hospital system was set up. At the township level and below, the system consisted of various clinics established through public-private partnerships, including union clinics, cooperative health stations, and individual private health practitioners (Table 1) [46]. The various models of provision at the township level and below were government-run district health stations and the above forms of providers with private or collective ownership. The guiding principle during this time period was to maximise the use of existing physical and human resources for health.
2. The People’s Commune Period (1958–1978)
  The three-tier system was established during the People’s Commune period (Fig. 7). This period was characterised by the communes becoming the primary government level in the rural areas. This was done through the integration of various previous township and village level agricultural cooperatives. Two main reforms took place. First, the different types of township level providers (government-run district health stations, union clinics, cooperative health stations and private practitioners) were centralized to communes. Second, the lowest level of the three-tier network was established at the village level as the prototype of the current village clinics, often set-up as pilots. These village level facilities were staffed with three types of community health workers: part-time health workers, first-aiders, and midwives [47]. The village level providers became responsible to record basic vital and maternal and child information, undertake health and hygiene promotion activities, provide epidemic control, and deal with minor complaints, participate in the management of the cooperative medical funds and refer patients to higher level providers [47]. The township hospitals were responsible for organizing and implementing disease prevention activities, providing basic maternal and child health care, including antenatal care, basic intrapartum care and postnatal care as well as acute hospitalization care for minor cases. The county hospitals were responsible for providing technical support to lower level providers in terms of epidemic prevention, infectious disease notification, comprehensive obstetric and child health care and treating patients that could not be treated at the lower levels of the system [41, 47].
  It should be noted that the health system development during this period experienced a number of constraints. During 1958-1964, when the central government centralized village level providers to township level, there were also unintended consequences. Since ownership for various union clinics, cooperative health stations and practitioners were centralized to communes, incentives to improve the quality and efficiency of health services deteriorated on one hand, while on the other, the supply of health care at village level suffered. As a consequence, the availability and accessibility of care in this period was actually reduced. However, this problem was addressed with the political endorsement of health care and the mass campaign of the Barefoot Doctor movement developed since 1965.
3. The Economic Opening and Decentralization Period(1978-present)
  Structural changes implemented during this period are shown on Fig. 8. The first change was the centralization of the administrative and professional management of township providers to county health departments. This time period oversaw the collapse of the rural health insurance system, i.e. the cooperative medical scheme (CMS) and the privatization of health care providers at the township level and those at the lower levels. In the early 1980s, the agricultural collective economy was replaced with a household responsibility system for production, in effect shifting the responsibility for economic activities from the state to local actors. This change was quickly followed by the drastic collapse of the CMS, as the system was deprived of its financial basis, and central government policies failed to provide a compensatory arrangement to the scheme [29]. As a result, village level providers became privatized and township and county level hospitals, health stations became autonomous. Instead of investment from the government and the collective economy, all level of providers became increasingly reliant on user charges. As a result, the relationship between different levels of providers changed gradually, from one of collaboration to one of competition. Furthermore, due to the decreased contribution from the government and the collectives, preventive care was gradually substituted by curative care which attracted higher user payments [11, 12]. These changes resulted in the collapse of the three-tier system in the 1980s. The new round of health reforms which has taken place since 2009 with the aim of reorienting the system to primary health care is yet to revitalize the three-tier system.

**Table 1:** Union clinics and Cooperative Health Stations

Union clinics
Union clinics were self-financed, for-profit facilities. Profits (after reserved funds for institution development and employee welfare) were shared according to private practitioners’ proportions of contributions. Such institutional structure was supported through political recognition in the 1955 National Culture and Education Conference: “Union clinics are social welfare institutions that are voluntarily organized by independent intellectual health practitioners”, "as small-scale collective ownership”.
Cooperative Health Stations
The cooperative health stations were primary health institutions established and financed by agricultural cooperatives. Physicians were members of the collective economy and were paid as normal farmers with mid-level working intensity through a system of “work points” to assess performance. Their main mission, similar to union clinics, was to provide primary health care. Profits from cooperative health stations were kept by the cooperative.

### Mechanisms

Having considered the policies that were at the core of the China’s progress to better health and expansion of coverage, and the political environment that gave rise to these, we discuss below the mechanisms through which these reforms were enacted, with various degrees of success, to establish the three-tier health service delivery system.

#### Diversified human resource development strategy

Responding to the extreme scarcity of health workforce serving in the rural areas, a human resource development strategy was introduced in 1951 [48, 49]. The policy consisted of three key strategies. First, medical education was reformed. Medical degrees were shortened to 4 years of college training and efforts were made to develop a 3 years secondary level medical training program.

Second, policies and campaigns were carried out to encourage the health workforce in urban areas to work in rural areas, for example as part of mobile medical teams. The Ministry of Health set itself a target to “allocate at least one third of the health professionals and administrative staff to serve in the rural areas” [45, 50]. The specific strategies included allocating two counties and their associated townships to each city hospital, and selecting the most talented health personnel to be deployed to live and work in rural areas [16–18]. In the first half of 1965 alone more than 12,000 health professionals from urban areas were recruited to take part in the mobile medical teams sent to rural areas. In addition, county hospitals sent over 17,000 health professionals to work in rural communities [9, 45]. By July of 1970, there was a total of 8000 health personnel serving as mobile medical teams [50–52].

Third, a large number of educated farmers, known as ‘barefoot doctors’, were recruited and trained for 3 months to be able to provide basic primary health care services in rural areas. This policy started in 1951, when the Chinese government issued a call for primary school teachers and members of the New Democratic Youth leaders with primary health training “without departing from their daily work and production activities, to conduct rural (township and village) health care activities” [48]. The policy was reformed in the 1960s, expanding the remit of barefoot doctors to improving hygiene and sanitation, performing acupuncture, providing care for “common ailments”, infectious disease prevention, emergency care, and delivery and basic birth attendance [45, 50]. The medical training of farmers during 1965 has been characterised as the “largest-ever medical training organized by the government in China’s history” [9, 16]. It played an indispensable role in the foundation and effective functioning of the three-tier health network in rural China. This new cadre generated substantial interest over the world, and has been described as “the health revolution that addresses rural health issues in low and middle income settings” [16, 33]. However, there has been debate on what can be considered comprehensive and appropriate primary health care, and evidence on the impact of barefoot doctors on health remains limited [9, 10, 24, 53].

These pragmatic solutions substantially mitigated the rural health workforce shortages in the rural areas and contributed to building the three-tier service network by strengthening the township and village levels of service delivery [7, 9, 21]. This had particularly important implications for improving coverage with primary health care services close to the communities.

#### Integrated approach to health service delivery

An integrated approach was a key mechanism for the effective implementation of the Chinese rural three-tier delivery network aiming to facilitate the government vision about achieving comprehensive and accessible PHC based on the ‘Health-for-All’ principles, for the whole population. There was a process of gradual and purposeful integration of the various levels of health administration, management and service delivery as discussed earlier, of preventive and curative services, and of traditional Chinese and Western medicine. This integrated approach ensured that many diverse institutions—governmental, civil society and medical associations—worked together within a unified policy and towards a common objective.

Mao publically endorsed the importance of traditional Chinese medicine and pharmacology are a “great treasure house… efforts should be made to explore them and raise them to a higher level” [7, 8]. By early 1950s, “uniting Chinese traditional medicine and Western medicine” had become an important principle of health system development. In this way, in the period of collectivization that lasted until the 1980s, in order to solve medicine shortages in rural areas, the population was encouraged to cultivate and produce traditional Chinese medicinal herbs [7, 9]. Importantly, this policy accommodated and legitimised a deeply rooted preference for using traditional medicines in the Chinese society, enabling it to develop often alongside mainstream medicine. Given the supportive policies from the higher policy-making levels of the health system, acupuncture and herbal medicine continued to be widely used in rural areas. The effective integration of Chinese traditional and Western medicine made a substantial contribution to responding to the extreme shortages of western medicines and technologies in the rural areas, thereby supporting the functioning of the rural three-tier health service network. For example, as noted by Horn (1972) “by 1971, over 400,000 operations had been performed under acupuncture anaesthesia with a success rate of about 90%” [7]. It is likely that acknowledging population preferences for traditional treatments maybe have also increased utilisation of the (often very basic) Western medicine.

System integration also stemmed from working toward the “Health-for-All” goal. This goal was pursued through a reliance on mobilising “the masses”, and incorporating health services with work and production [37].

#### Innovative financing mechanisms

Successful policy implementation was also enabled by the adoption of innovative health financing strategies. Reflecting the political commitment to rural health, salaries of the national employees and barefoot doctors serving the rural three-tier health services network were paid by the government in the first two historical periods before 1978. For example, in 1973 the state pledged to subsidize “collective (commune) hospitals by 35% of the expenditure of the hospital (except for pharmaceuticals and medical materials) or 60% of the salaries of the collective” [54].

However, due to fiscal constraints, the daily operations of township health stations remained reliant on user fees. An extreme lack of affordability among the rural populations is likely to have hampered the revenue of the rural health providers and viability of the services [36, 40]. To respond to this situation, two innovative financing approaches were implemented to provide support to rural health facilities in raising and maintaining their operational funds.

The first is the gradual expansion of the Cooperative Medical Schemes (CMS); a rural community health insurance system founded on the principle of mutual cooperation. With the support of government and the rural collective economy, rural residents pooled funds at the village or township level to cover health care costs. The CMS was piloted in 1959, and was gradually introduced and adapted. By 1968, extending the CMS was the core health policy in rural areas, with pooling of funds being gradually centralized from brigades to communes [16, 55–57]. By 1976, 90% of the brigades had set up a CMS. [56] Brigade clinics and commune hospitals managed the CMS funds, and in order to get financial reimbursement, patients were first required to visit a commune hospital (or below) before receiving a referral to higher level providers [16]. Thus the CMS became the main financial source in supporting the running of township and lower level health institutions [56].

The second financing mechanism was a modification to the industrial registration policy, which exempted all types of health facilities from paying tax. This tax exemption policy was set up in 1950, and allowed health providers to retain any revenue they raised and reinvest it with the aim of removing financial barriers to provision of health services. The policy encompassed all types of providers—including private, collective or public private health providers, including Chinese medical practitioners—on condition that they committed to providing medical services (including free services for the army), epidemic prevention, maternal and child health care services and provided a proportion of inpatient and outpatient services for free. In addition, providers were required to charge rates for health services set up by local health authorities [58, 59]. This policy was considered essential to alleviate financial shortages by the experts validating the findings.

#### Public-private partnerships

As a response to the fiscal constraints and the broader political and economic context, the rural three-tier system adopted a system of public-private partnerships and diversified ownership models in order to accelerate the establishment of rural townships and village-level health care providers. This strategy started during the Agricultural Collectivization Period (1949–1957), when the government was pursing the objective of building one hospital in each county [38, 46]. For health facilities at the township level and below, the government identified lack of resources as the key obstacle to expanding essential care, and in response, private-public partnerships were promoted [48, 49, 59, 60]. In addition to government-run district health stations, quasi-public health providers such as cooperative health stations, and private providers–such as the union clinics (Table 1) were encouraged to practice as individual practitioners or pharmacies [38, 47, 48, 56, 61]. Reforms varied according to the degree of decentralization and the size of townships [16].

This policy was later abolished during the People’s Commune Period (1958–1978), which saw the establishment of unique collective-run township health stations for each commune, with three types of community health workers in each village [62]. All health service providers at the township level and below were merged to establish township government-run health facilities, which were known as commune hospitals and were equipped with 8–15 personnel and 1 bed per 1000 population [47]. Once this process was completed, all individual and private practices, including pharmacies, were abolished. Supplies and funds were considered as investment to the communes and then transferred to collective ownership. The commune covered food expenses as well as health worker salaries [44, 63].

Since resources were centralized to township level, there were no providers any longer providing services at the village level. Although the objective of merging resources was met, access to health services decreased, as patients would have to travel long distances to reach the townships [16, 33]. All assets belonged to the commune collective, though some stations were owned by the villages and brigades; overall guidance was provided by the commune (township level) health care stations [41].

After 1978, during the Economic Opening and Decentralization Period, both the CMS and the rural collective economy collapsed. Rounds of debates took place, and comprehensive reforms have been put forward since the 2000s, with a particular emphasis on the rebuilding the delivery networks and revitalizing the rural three-tier health system [64]. A key focus for the government is to fully cover the township providers’ salaries and operational costs, and reduce their dependency on user fees. The process of establishing township health stations as operational and publicly owned facilities continued until 2011. Since 2014 reforms have been introduced to further include village clinics into the core public sector network and work towards their integration [14, 65].

#### Flexible policy implementation

The foundation and development of China’s rural health system was marked by the adaption and gradual modifications of reforms; this may have been an important mechanism for ensuring their effective implementation. This approach can be demonstrated in two areas of policy development. A flexible and adaptive policy development approach was used in the establishment and development of the rural primary health facilities. Various forms of ownership were tried and abolished as context changed, promoting the coordination of funding and human resources, as well as improving the work motivation of health providers working at all levels. As the first step, through the government’s direct investment, existing resources were fully integrated to establish the county hospitals [66]. Townships which were able to do so were also encouraged to build district clinics [67]. As a second step, and in response to the fiscal constraints experienced by health providers at the township level and below, the government encouraged different types of private providers to participate in order to extend service coverage. Finally, after the establishment of the township level providers, which since 1958 included district health centres, union clinics, cooperative health stations, private individual practitioners, were integrated into commune health care stations. After the “June 26th directive” [68], a large number of urban health professionals were sent to the countryside to help build rural health service network. The collective ownership were strengthened at the township level. “Rural commune health care stations…… should be gradually transformed into organizations that are owned and run by commune or production team.” However, since the move towards increased autonomy of facilities during the 1980s and 1990s, the government has reconfirmed its obligations for direct financial support and control of township providers since 2009.

Another areas in which the Chinese government employed an adaptive and realistic human development strategy in response to the extreme shortages of health workers [7]. Community health workers were mobilized to serve in the village clinics, training courses were shortened for doctors who worked in the county and township hospitals, mobile teams were sent from the cities in rounds of mass campaign movement to serve in the rural and carry out on-site trainings. It was documented that during the 1960s and 1970s “the training of part time health workers, should abide by the principle of less teaching but more practicing and learning by doing……they may be trained by the mobile medical teams or health professionals from local health facilities, they could also be trained in the medical training classes in the local middle schools. The rural doctors were also trained during times when agricultural work is not intense and return to agricultural work in the cultivating and harvesting time. They were trained in basic knowledge and skills to deal with common ailments for 3 months by doctors from county and higher level hospitals, and were expected to go back to serve in their village. Undertaking 2 to 3 years’ continuous training in such a format typically enabled health professional to obtain accreditation” [41]. In practice, the training of rural doctors was practical” normally half a year training and half a year practicing” [41]. With continuous training, they could normally achieve secondary education level in 2 to 3 years [17, 18, 67]. As a result in just 1 year, a large number of barefoot doctors were trained by the mobile medical teams, who were assigned to build up village health stations and consolidate and strengthen township hospitals.

### Outcomes

The ToC underpinning this research sought to identify also the outcomes associated with reform implementation—in the area of improved health, access to essential services and building of effective service delivery systems. Apart from the conceptual difficulties of linking inputs and implementation modalities with outcomes, the analysis is handicapped by the lack of publicly available data, due to the destroy of the Cultural Revolution on government archives [33], on health and access to care during many of the historical periods; thus we mainly refer to outputs, process indicators or intermediary outcomes. Many of these related to the capacity and operation of the health system. However, it is important to note that the section synthesises the findings drawing on the perspectives of the authors whose work is included in the paper. The indicators used in different periods, and the importance attributed to each, vary.

#### Health system development

The outcomes that are associated with these reform developments are mainly seen in strengthening the delivery function as a fundamental block of the health system: a network of rural facilities served by trained and present workforce providing accessible and affordable care appropriate to context. The ability of the health system to provide a range of essential health care was strengthened. This included curative services, maternal and child care (including antenatal care, comprehensive intrapartum care and postnatal care-, child growth monitoring and immunization).

Considerable capacity for large-scale epidemic prevention and control, environmental hygiene was reported [8, 9]. Many initiatives benefited from a cross-sectoral nature, such as health education and improved access through community awareness of services. There were considerable benefits in other ‘blocks’ of the health system—thus, information collection and reporting was expanded in line with the newly created structures [16].

At the same time, the structures and processes within the health system were developed—including trained and deployed workforce, administration and managerial cadre. The integrated nature of the services encouraged networking and referrals between the different levels. Advances were seen in developing effective governance structures, and their ability to plan and deliver sets of complex policies while allowing for experimentation and frequent adjustment.

As a result of substantial government investment, a number of county hospitals were established by government investment and the integration of various existing resources; by 1952, the policy of having one county with one county hospital was implemented in 90% of all counties nationwide [40]. According to official statistics, in rural China, hospital beds per 1000 population increased by eight-fold from 0.1 to 0.8 per 1000 population till the middle of the People’s Commune Period [69]. During the same time period, the number of health professionals formally employed by township and county hospitals also increased from 1.1 to 1.5 per 1000 population.

There was also a rapid development of township health facilities. By 1957 central government documents reported there were over 50,000 union and township clinics and about 10,000 health stations established by agricultural cooperatives, together employing about 200,000 health professionals nationwide [43]. Health worker at primary level were extremely diverse suggesting using realising approaches to fill the gaps: including individual private health practitioners, pharmacy based physicians, and part-time health workers, part-time health workers in Red Cross organizations at primary level, farmland community health stations and delivery stations.

The analysis suggested that the three-tier system has led to development of the intelligence and information health systems block, building extensive health information systems. All health institutions within the rural health service delivery network were responsible for collection of coverage data and reporting epidemics, and accountable for their activities to the higher bureaucratic levels. Conversely, the network model and strong vertical and lateral linkages within the rural three-tier health service meant that administrative instructions from the central level could easily reach frontline organizations. Equally, information provided by grassroots organizations could be easily summarised and used by the national level planners to monitor and evaluate reforms, and their continued refinement. Thus despite that no information technology was available, a national epidemic reporting system was established in the early 1950s, capable of reporting infectious disease epidemics within 3 days [16].

#### Health service utilisation

The rural three-tier health service delivery network not only transformed rural health care, but was also perceived to meet the medical needs of rural residents during its expansion. Coverage of clean delivery—equivalent to skilled birth attendance [33, 69, 70]—increased to 61.1% in 1957 and 91.4% in 1980, which was seen as a remarkable achievement in terms of improving the maternal and child health outcomes [33, 69, 70].

**Fig. 9:**
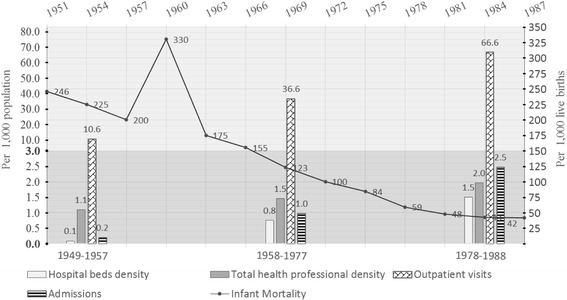
Trends in health system resources, health care utilizations and infant mortality in rural China during 1949–1988

The increasing availability of resources corresponded with a great improvement in access to health services. For example, utilization of outpatient care tripled, and admission rates increased five-fold in rural China between 1949 and 1977 (Fig. 9). By the early 1970s the three-tier health service network was fully developed and could manage “minor illnesses revolved within the production team, moderate illness within the commune, and major illness within the county” [16]. This period was recognized as the “15-year honeymoon of the state and farmers” in China [16, 56, 71].

#### Health outcomes

**Fig. 10:**
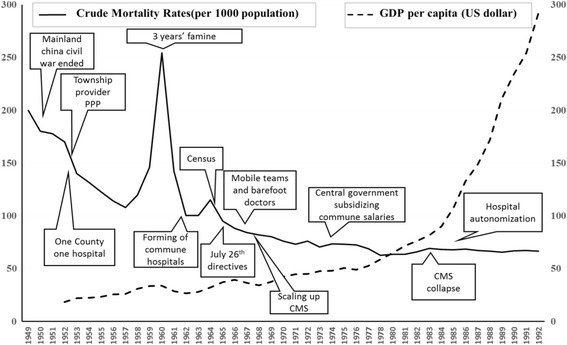
Crude mortality and the development of three-tier system in rural China

The country initiated reconstruction in 1949 with extremely scarce resources in a context of very poor socio-demographic characteristics. Life expectancy was 35 years on average, there was a high prevalence of infectious diseases, and high levels of maternal and under-five mortality (in 1949 maternal mortality was estimated to be 1500 per 100,000 live births and infant mortality 200 per 1000) [2, 7–9, 30]. The key policy initiatives and phases are mapped against the crude mortality rate for illustrative purposes (Fig. 10). This suggests that the improvement in health outcomes does not appear to be associated with increasing national wealth.

As noted by Dr. Keane, a former WHO representative: “China’s health system has made remarkable achievements. If you look at statistics like life expectancy, infant mortality, causes of death, etc. You cannot believe that it is a developing country” [40]. For example, from 1949 to 1977, infant mortality decreased from 246 to 48 per 1000 live births [70] (Fig. 10) The country’s census in 1964 reported life expectancy increased to 67.9 years of age (male 66.4, female 69.4) [2] and the maternal mortality rate reduced to 48.8 per 100,000 live births in 1984 [72].

## Discussion

The creation of a three-tier health service delivery network in rural China in the 1960s was a result of substantial political, financial and bureaucratic investment and long-term policy efforts. It delivered an accessible and community-owned service, involving a de-professionalized workforce providing low tech, economically feasible and culturally appropriate services, despite large-scale poverty and socio-economic constraints. The system was considered as a success story in providing essential health care services to the resource-scarce settings in terms of its ability to provide a wide range of health services at low cost, whilst managing strong health information systems and surveillance despite a basic technology, and to achieve health improvements on a wider scale.

The conceptual framework was used as a tool to identify key policies, mediating factors, pathways and outcomes and to derive a narrative, as well as to assess the transferability of the Chinese health system development model to other settings. The analysis was underpinned by a rigorous search strategy, capturing a wide variety of study designs, policy and administrative documents located in public and government archives, doctoral dissertations and journal articles. Findings from the review were triangulated against analysis by key experts, intending to capture a panoramic view of the development of the three-tier system over time. However, the analysis was hampered by the lack of accessible information on earlier periods even in grey literature in China. This was particularly the case for health outcomes where existing analyses are almost entirely lacking, and access to state statistics and routine data is problematic. In order to construct as comprehensiveness picture as possible, the synthesis included a wide variety of documentary sources providing information relevant to the research questions. This was accompanied by efforts to maximise triangulation and validation of information through consultation within the extended international team and with experts who have played key roles in the reforms, and testing findings against the ToC. This process demonstrated a high level of consistency among the findings within the different dimensions of the ToC framework: context, policy contents, mechanisms and outcomes. The study found no clear evidence quantifying the effectiveness of the health strengthening efforts in improving population health for China during the early two phases of the development of the three-tier delivery network. Given the lack of data on outcomes, the study used process indicators to examine how the health system strengthening efforts across different dimensions may have contributed to particular health outcomes.

Our findings show that China’s three-tier health service delivery system was designed to respond to a challenging health situation in a vulnerable nation emerging from conflicts and high level of poverty and inequality: high fertility, high mortality due to infectious diseases and maternal and child conditions, high maternal and infant mortality and low life expectancy, where health system resources were unable to respond to this high burden of disease [2, 7]. Six decades ago, with strong political commitment and effective leadership prioritising rural health and relying on mass campaigns, a three-tier (village-township-county) health service delivery network was rapidly established and scaled up. This study suggests that these policies achieved their intended objectives through six programme mechanisms: a diversified and pragmatic human resource development strategy, an integrated approach to health service delivery, innovative financing mechanisms, public-private partnerships, an emphasis on prevention, and an integrated approach to provide essential health services, including the integration of prevention and public health with curative care, and the integration western medicine and traditional Chinese medicine [16, 40]. The considerable structural and process integration and coherence across health systems levels and structures facilitated planning and administration. The government made strong commitments and investments to fulfil its “one county with one county hospital” objective as pledged by the central political leaders [38, 46]. To achieve this objective, private-public partnerships in township and lower levels were encouraged to fill the resource gaps, comprising diverse providers, including union clinics, agricultural health station and private practitioners. Together with other support, including the cooperative medical scheme and the tax exemption policy, the rural three-tier system was quickly established in the People’s Commune Period (1958–1978), integrating the various private providers into a united government-run system. To increase efficiency, planning and allocation of resources was undertaken at the administrative level, with an emphasis of having people-centred health services. Finally, adaptive policy implementation and capacity for incremental change have been important mechanisms ensuring policy objectives are met.

The evolution of the three-tier system has clear parallels with the development of the Semashko model in the former Soviet Union, also replicated in central and Eastern Europe (1945–1989). Both models developed health care delivery networks staffed by midlevel and auxiliary cadre rapidly expanding geographic access to PHC over often very large areas [73]. It involved a strict hierarchy and vertical and horizontal integration across different levels of the health systems, enabling effective referral to key essential secondary level. In both models there was an integration of curative, preventive and public health services [74]. The Soviet Semashko model was highly hierarchical, involving higher levels of authority formulating policies which were then operationalised and implemented by lower levels with tight accountability, with similar structure seen in China. The key difference is that the Soviet Semashko model entailed a universal entitlement to a comprehensive package of publicly financed and delivered health care for the majority of the population while in China the rural delivery model incorporated private providers and out of pocket payments. However, after the political changes in the 1980s, the Soviet Semashko model was mostly abandoned in terms of its core goals, structures and linkages, with limited features of the system surviving the transition to a market economy. Both models services experienced deterioration of rural PHC facilities, perception of low quality of PHC and preferences for seeking specialist care even at considerable cost to users. The Chinese version, however demonstrated a higher degree of integration, e.g. involving public and private providers as require to fill gaps in coverage. Another important difference was the considerable flexibility for policy adaptation and incremental change which strengthened policy implementation in China, recognising the regional diversity and need to allow autonomy in reform experimentation. In contrast, in the former USSR, efforts to decentralise health systems resulted in poor accountability and political tensions [75], with pilots of new initiatives implemented only in some of the countries and less often integrated into policy cycles. Use of intelligence was also markedly different, in China reforms developed through series of pilots producing evidence of coverage and effectiveness of particular initiatives, while the USSR model often failed to utilise new evidence and promoted isolationism [73].

The experience of China over the 60 years of health care delivery development provides useful lessons for other LMICs seeking to establish and operate a rural health service delivery network providing essential PHC despite constrained resources. Our analysis suggests that even when China suffered extreme shortages of medicines, technologies and skilled health workforce, strong governance embedded with people-centred and health-in-all perspectives, a de-professionalized, community-oriented, and culturally appropriate health care delivery model helped to extend essential services. This experience also helps to identify mechanisms in which these policies operated and ways in which the problems were overcome. Importantly, while policy content is unique to each setting, it has been argued that the programme mechanisms are potentially transferrable to other settings [76]. Lessons can also be learnt from China in centralizing and transforming ownership of various village level providers and forming commune health care stations. However, health system development rarely follows a linear pattern from cause to effect. Policy implementation is shaped by targeted policies but also the sociopolitical context. It is a gradual process, adaptive to socio-political change and stakeholders’ reactions. However, there are almost no empirical and analytical studies from this period, and this studies draws on information obtained from official policy documents; we recognize this as a limitation of this study.

The features of China’s three-tiered delivery systems during the earlier periods may shed light on China’s current efforts in strengthening its primary health care. Collaborative, coordinated, comprehensive and continuing care could be offered by forging a strong primary care system linking patients, families, communities and health care organizations. However, as this paper suggests, it is important to take into account contextual factors. Before the 1980s China had low health expenditure, and low intellectual, technological and medical capacity; therefore the quality of care provided at the primary level was very basic. With rapid economic development, population demand for health has increased substantially, and some of solutions of the past may not fit with the new realities. For example, the flagship barefoot doctors movement which was credited with the improved access to PHC in rural areas in the 1960s–1980s, may no longer fit with population expectations and perceptions that the quality of care provided by these “less skilled” primary care providers is not and cannot be improved, and would prefer instead to seek primary health care in large hospitals. Thus if designing a new model of community health providers, it is important to rethink their skills profiles, role and links with hospitals, in the framework of a strategy for development and improvement of human resource for health. While the Chinese experience demonstrates the benefits of a community-oriented delivery models, it also shows how gains are reversible within a relatively short time period [77, 78].

Since 1978, the central government decentralized health care financing and the cooperative medical schemes collapsed in line with the transformation of the nation’s rural economy from an agricultural collective system to a household responsibility one. Consequently, providers’ relationships changed gradually to competition rather than collaboration, ushering in the fragmentation of China’s health care system in providing quality preventive care and primary care. When the government cannot guarantee financial support for PHC, incentives of primary health care providers can be distorted, the function of the delivery network can be undermined and a primary health care system may be threatened once the collaborative and accountability relationships have broken down. However, the continued drive to adapt and fine-tune policy reflects an understanding of health system development as a process of incremental change and building on its inherent path dependency [79]. Understanding this model of development, both of terms of its content and the process through which it was implemented, and the institutional and contextual factors supporting it, provides useful lessons to other LMICs.

## Conclusions

China’s experience in establishing a de-professionalized, community-centred, health service delivery model that is economically feasible, institutionally and culturally appropriate mechanism to deliver health care in rural areas can provide useful lessons to other LMICs seeking to extend essential services. Preconditions for success of the three-tier delivery model were created through series of policies developed over a long period of time (1949-1980s) and relying on shared values of collective responsibility of health and local accountability. This experience also shows how gains can be reversed in a short time period after the supportive societal and health systems structures were dismantled. Understanding how this model has developed in its unique socio-political context is key. However, lessons can be drawn from both reform content and from its implementation pathway, identifying the political, institutional and contextual factors that have shaped it. Once these are taken into account, aspects of policy content and process may be relevant and transferable to other settings. Learning from the evolution of the three-tier delivery model is particularly important also for China as it seeks to revitalize its primary care system and ensure it is fit for a new era.

## Abbreviations

CMS: Cooperative medical scheme
CPC: Communist Party of China
LMICs: Low- and middle- income countries
PHC: Primary health care
ToC: Theory of change
USSR: Union of Soviet Socialist Republics
WHO: World Health Organisation
